# Accessible Biofabrication of Anatomically Inspired Hollow and Branched Hydrogel Constructs by Soft Templating (Sof-T)

**DOI:** 10.3390/bioengineering13070838

**Published:** 2026-07-21

**Authors:** Jacob Dairaghi, Dominic Joseph, Horia I. Petrache, Nicanor I. Moldovan

**Affiliations:** 1 Department of Biomedical Engineering, Purdue University, Indianapolis, IN 46202, USA; 2 Department of Physics, School of Science, Indiana University, Indianapolis, IN 46202, USA; 3 Department of Surgery, School of Medicine, Indiana University, Indianapolis, IN 46202, USA

**Keywords:** CAD, biofabrication, 3D printing, bioprinting, alginate, crosslinking, anatomic realism

## Abstract

Biofabrication has significant potential to advance medicine and research by creating complex and anatomically accurate engineered tissues for implantation or in vitro modeling. However, a persisting challenge of the current biofabrication methods, such as hydrogel-based bioprinting, is to find an efficient, affordable, and reproducible method for the generation of hollow and branched geometries. This is critical for the recapitulation of anatomically realistic structures representative of cardiovascular, respiratory, and other organ systems. Existing bioprinting approaches require complex, multi-step processes and expensive specialized equipment, limiting accessibility and extending fabrication time. Here, we present an alternative ‘sacrificial’ method for the rapid and accessible creation of hollow and/or branched hydrogel constructs, which we term ‘soft templating’ (Sof-T). Sof-T utilizes ionic diffusion from a 3D-printed water-soluble polymer to crosslink surface-adsorbed hydrogels followed by the dissolution of the polymer, thus leaving behind the anatomically patterned hydrogels. Using this technique, we readily generated: (1) vascular-like bifurcated aortic conduits, with or without aneurysmal deformities; (2) upper and lower (branched) trachea models; and scale-reduced (3) human hearts and (4) bladders. Overall, Sof-T offers a simple, rapid, and cost-effective strategy for fabricating relatively complex, hollow hydrogel architectures, broadening the access to anatomically relevant constructs for biomedical research and translational and/or educational applications.

## 1. Introduction

Tissue engineering and three-dimensional (3D) bioprinting represent transformative approaches in regenerative medicine, offering potential solutions to the critical shortage of donor organs and tissues [1,2,3]. The field has evolved significantly since early developments in composite scaffolds for engineering hollow organs and tissues [1], with researchers exploring various biomaterial combinations to overcome the limitations inherent in individual scaffold types [4,5]. Bioprinting has specifically gained considerable attention as a potential method for creating biological tissues, raising the possibility of reproducibly creating complex macro- and microscale architectures using multiple different cell types [6,7,8]. Therefore, it is a promising method for the creation of multilayered hollow organs that have been challenging to make using more traditional tissue engineering techniques [2,9].

For example, the bioprinting of blood vessels and heart structures has advanced rapidly, though current capabilities remain limited to a specific selection of biomaterials [10,11,12]. Recent developments in scaffold design have successfully employed porous and fibrillar architectures to address the layered structure of cardiac tissue [3,13], while the availability of induced pluripotent stem cell-derived cardiomyocytes has opened perspectives for creating larger functional constructs [4,14]. Using these cell sources, researchers have made scale-reduced heart models through bioprinting techniques [4,15], though full anatomical-scale models remain limited to acellular constructs, specifically those based on collagen matrices [5,16]. Recent advances in embedded 3D bioprinting have expanded the design repertoire for fabricating geometrically complex tissue scaffolds using hydrogels with mechanical properties comparable to native tissues and organs [5,17], demonstrating the ability to bioprint full-size models of adult human hearts from patient-derived magnetic resonance imaging data sets [5,18].

Alginate, either alone or combined with nanocellulose, has emerged as a highly versatile bioink material due to its biocompatibility, printability, and crosslinking properties [19,20,21]. The use of sacrificial materials has made many of these advances possible, with the most commonly used being Pluronic [6,22]. Alternative approaches include sugar-based glasses [23] and polyvinyl alcohol through techniques such as Negative Embodied Sacrificial Template 3D (NEST3D) printing [7,24]. NEST3D printing demonstrates how negative patterns within three-dimensional printed water-soluble templates can describe geometries that are extremely challenging to print directly by extrusion, creating complex three-dimensional structures including hyper-branched dendritic shapes and open lattices with tunable stiffnesses [7,25].

Despite these advances, current bioprinting approaches continue to face fundamental limitations that restrict their widespread adoption [26,27]. The extrusion process subjects cells to mechanical shear stresses that can compromise viability and function [28,29], while the layer-by-layer deposition process is time-intensive for large constructs [30]. Furthermore, the specialized equipment required for bioprinting is expensive and requires technical expertise that may not be readily available in all research settings [31,32]. These limitations have created a clear need for alternative biofabrication approaches, such as Sof-T, which can achieve similar outcomes while utilizing more accessible methodologies and equipment.

The specific innovation of our method is not the use of ‘sacrificial’ water-soluble templates per se, but their use as self-crosslinking templates in a one-step diffusion-and-adsorption process, therefore: (i) unlike Pluronic-based sacrificial bioprinting [22], Sof-T does not require print-in-print co-deposition; (ii) unlike NEST3D [7], which produces the crosslinker/hydrogel geometry by extrusion within a sacrificial matrix, Sof-T uses the sacrificial template itself as the ion reservoir and shape-defining surface, allowing the hydrogel to take the desired form by diffusion rather than extrusion; and (iii) unlike embedded FRESH bioprinting [10], Sof-T does not require a support bath.

The successful fabrication of vascular bifurcations, aneurysmal models, and scale-reduced cardiac and bladder constructs shown here demonstrate the versatility and potential of this approach across a range of tissue engineering applications. The dimensional accuracy and structural fidelity achieved, combined with the accessibility of required equipment, suggest that the Sof-T method could serve as a practical alternative, at least in some applications, to more expensive and/or complex fabrication techniques.

Our technology’s requirement for only standard 3D printing equipment and readily available materials represents a significant step toward increasing the accessibility of advanced biofabrication capabilities. This could contribute to research progress in tissue engineering and regenerative medicine, by enabling more laboratories to participate in developing and testing complex tissue constructs.

Future work will focus on refining process parameters to achieve more uniform wall thickness, implementing dual crosslinking systems for improved mechanical properties, incorporating cellular components for functional tissue constructs, multi-layered construct fabrication, and expanding applications to additional tissue and organ systems. These developments may help establish the Sof-T method as a valuable complement to existing biofabrication technologies and will likely enhance the utility and impact of this approach in research and, with more validation, possibly in clinical settings as well.

## 2. Materials and Methods

***Three-Dimensional Printing of Water-Soluble Polymers.*** Polyvinyl alcohol (PVA) or butenediol vinyl alcohol (BVOH) copolymer was 3D printed using either Ultimaker 3 (Ultimaker BV, Utrecht, The Netherlands) or Ender 3 (Creality, Shenzhen, China), respectively. PVA has a lower glass transition temperature and strong hydrogen bonding than BVOH, meaning the polymer remains in a viscoelastic state longer before cooling. BVOH is a modified copolymer that reduces hydrogen bond interactions and solidifies more quickly, which can improve dimensional accuracy and ease of use in 3D printing. In addition, PVA has higher moisture sensitivity, which may reduce printing quality, and that guided our decision to switch to BVOH as a preferred water-soluble 3D printing filament.

Filaments with a diameter of 1.75 mm of either polymer were printed at a temperature of 210 °C, with a layer height of 0.2 mm, and a print speed of 40 mm/s (occasionally adjusted lower for more complex geometries, to increase detail and success rate of print). The infill was 15% and bed temperature 90 °C with adhesion.

***Hydrogel construct generation.*** In brief, alginate solutions with different concentrations were prepared as previously described [33]. For crosslinking we used 10% (*w*/*v*) of either calcium chloride (CaCl_2_) or barium chloride (BaCl_2_) solutions in distilled water. Printed templates were soaked in CaCl_2_ or BaCl_2_ solution from 10 min up to 1 h depending on the size of the template, to allow ion penetration throughout the architecture. To remove the templates, after a brief immersion in alginate for adsorption, the constructs were placed in warm water with magnetic stirring for template removal for 1 h up to 24 h, depending on size. For the Sof-T method presented here, we followed the protocol detailed in the Supplemental Data; see also [33,34,35].

***Structural and Mechanical Characterization.*** Alginate constructs that were 10 mm in diameter were fabricated for structural characterization and to assess suitability for some of the intended applications. First, the models were evaluated for wall thickness uniformity using digital calipers and closer examination by optical microscopy, or by comparison with original CAD design, and structural integrity was assessed through uniaxial tensile mechanical testing. Perfusion testing was conducted using colored water to verify the patency of internal lumens and the absence of structural defects.

## 3. Results

### 3.1. Sof-T Fabrication of Perfusable Alginate Tubes

**Figure 1:**
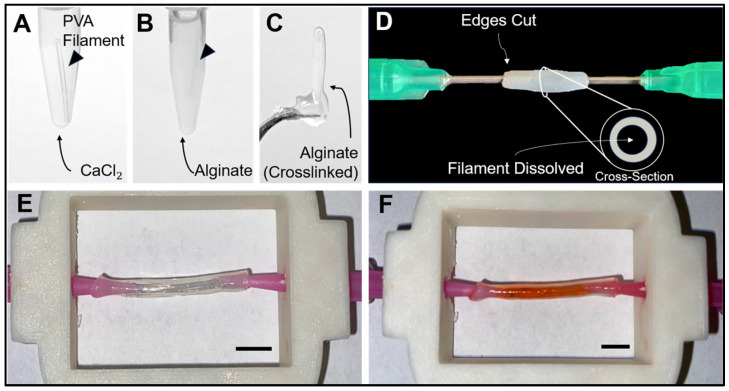
Sof-T generated, perfusable alginate tubes. (**A**) A PVA filament was soaked in a calcium chloride solution, then (**B**) submerged in a sodium alginate hydrogel, and (**C**) crosslinked by Ca ions diffusing from the template. (**D**) Resulting alginate tube after trimming and dissolving the PVA filament cannulated at both sides with syringe needles. (**E**) Fabricated alginate tube attached to a pump before and (**F**) after perfusion with a red-dyed solution. Scale bars: 1 cm.

To test the Sof-T protocol, a 3D printing-grade polyvinyl alcohol (PVA) filament segment was cut and incubated in a chloride (CaCl_2_)-based ionic crosslinking bath for 10 min, where the polymer absorbed the ionic crosslinker throughout its entire length (Figure 1A).

The ion-loaded filament was subsequently placed in a 4% *w*/*v* sodium alginate hydrogel for 5 min, thereby inducing ionic crosslinking at the interface of the polymer (Figure 1B,C). After crosslinking, the ends of the construct were trimmed to reveal the PVA filament. Incubation in room-temperature water was used to dissolve the PVA, resulting in the formation of a hollow alginate tube (Figure 1D). Such freestanding tubes could be safely manipulated to be placed within a perfusion chamber (Figure 1E). Perfusion of a red-dyed solution showed no leakage onto a white paper background, demonstrating that these structures retain the fluid during simple perfusion (Figure 1F). For additional details, see the extended protocol in the Supplementary Information.

### 3.2. Optimization of Alginate Concentration for Structural Fidelity

**Figure 2:**
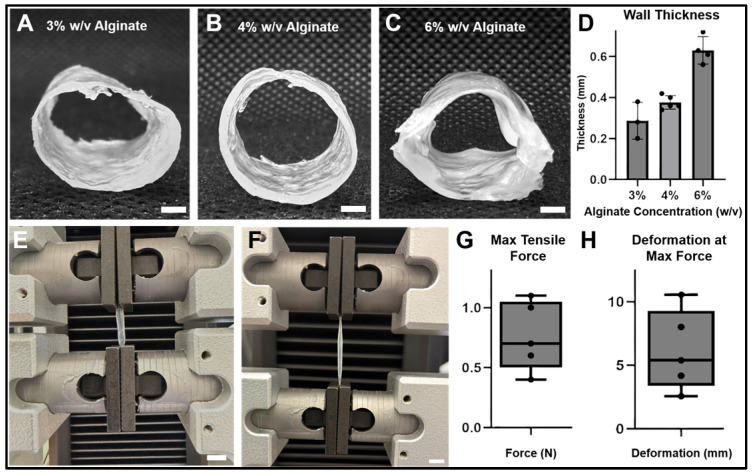
Optimization of construct resolution and mechanical strength. (**A**–**C**) Alginate tubes fabricated using either 3%, 4%, or 6% alginate, respectively. (**D**) Thickness of tube walls. (**E**) The 4% *w*/*v* alginate tube either before or (**F**) after mechanical stretching. (**G**) Maximum tensile force and (**H**) maximum deformation of 4% *w*/*v* alginate tubes (*n* = 5). Scale bars: (**A**–**C**,**E**,**F**): 1 cm.

Alginate concentration is critical to successful fabrication by the Sof-T method. To compare differences between alginate concentrations, cylindrical templates were 3D printed using BVOH as an alterative water-soluble polymer. After saturation with calcium ions as described, varying alginate concentrations (3%, 4%, and 6% *w*/*v*) were adsorbed to these BVOH cylinders by subersion, then retrieved for template removal. The resulted crosslinked alginate tubes were then examined morphologically from a lateral perspective (Figure 2A–C).

The samples prepared with 3% *w*/*v* alginate concentration showed significant sag and deflection under their own weight (Figure 2A), indicating that these structures were not stiff enough to maintain the intended shape, i.e., in this case to remain cylindrical. Instead, the tubes produced using the 4% *w*/*v* alginate showed good fidelity to the template, with well-defined circular cross-sections and no deflection from the cylindrical structure (Figure 2B). The structures produced using 6% *w*/*v* alginate were also stiff but showed poor post-adsorption fidelity to the template, showing irregular non-circular cross-sections, likely due to the increased viscosity of the alginate precursor solution (Figure 2C). Alginate concentration was directly related to wall thickness, with 6% alginate having the thickest walls at 0.63 ± 0.07 mm, and 3% and 4% having similarly thin walls at 0.29 ± 0.09 mm and 0.38 ± 0.03 mm, respectively (Figure 2D).

To further characterize the mechanical properties of the optimal constructs, 4% *w*/*v* alginate tubes were also subjected to uniaxial tensile testing (Figure 2E). To this end, these hydrogel tubes were placed between the grips of a mechanical testing device and subjected to increasing longitudinal tension until failure, with a large degree of elongation visually evident before breaking (Figure 2F). The maximum tension at failure over five samples was an average of 0.76 ± 0.29 N (Figure 2G), and the degree of stretching at maximum tension was an average of 6.1 ± 3.2 mm (Figure 2H), indicating that these tubular constructs have sufficient elasticity and resistance to withstand moderate mechanical manipulation and physiological stretching without premature failure.

### 3.3. Fabrication of Complex Anatomically Inspired Geometries

**Figure 3:**
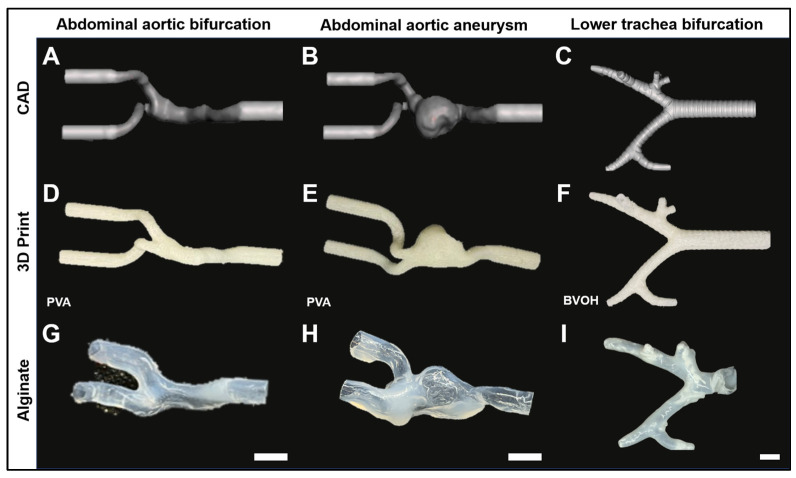
Fabrication of branched hydrogel constructs using the Sof-T method: (**A**–**C**) CAD model, (**D**–**F**) 3D prints with water-soluble polymers, and (**G**–**I**) resulting alginate construct for: abdominal aortic bifurcation, an abdominal aortic aneurysm, and a lower tracheal bifurcation. *Notes:* (1) hydrogel tubes ends were trimmed for template solubilization; and (2) positions for imaging of hydrogel constructs could match that of corresponding templates only approximately. Scale bars: 1 cm.

With the optimal parameters determined using simple geometries, we sought to generate increasingly complex geometries using the Sof-T technique. To achieve this, three complex human anatomical geometries that contained branching structures were selected: aortic bifurcation, aortic abdominal aneurysm, and lower tracheal bifurcation. Computer-aided design (CAD) models were created to represent each anatomy (Figure 3A–C). Following this step, the corresponding templates were 3D printed using either PVA (Figure 3D,E) or BVOH (Figure 3F). Then, the next steps of our Sof-T technique were used to produce hollow alginate replicas of the templates (Figure 3G–I).

**Figure 4:**
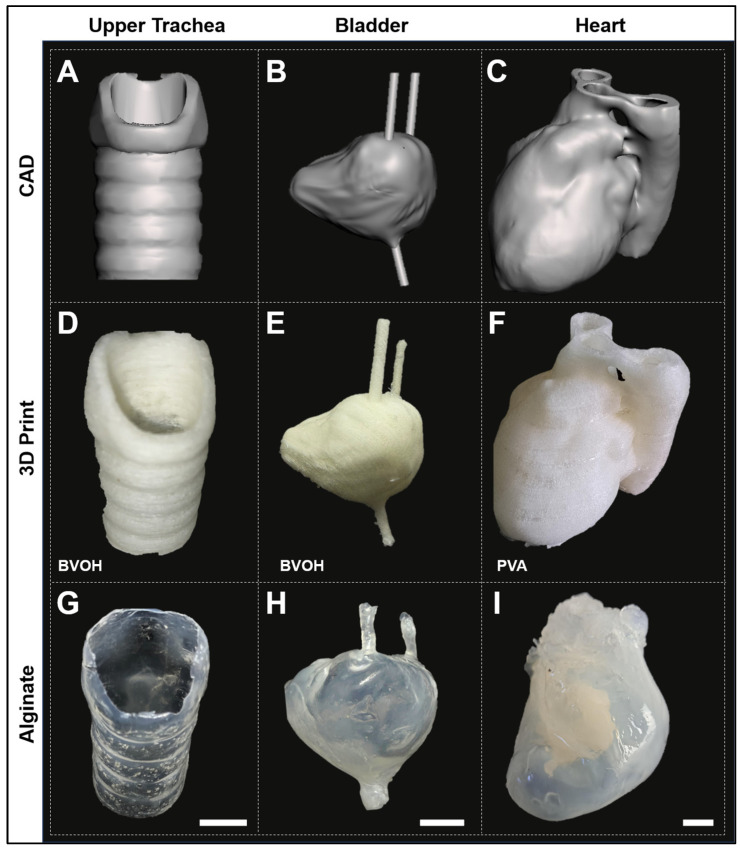
Fabrication of anatomical hollow hydrogel structures using Sof-T: (**A**–**C**) CAD models, (**D**–**F**) water-soluble 3D prints, and (**G**–**I**) resulting alginate constructs. Scale bars: 1 cm.

The versatility of the Sof-T technique was further demonstrated by the fabrication of even more complicated hollow geometries that mimic the internal architectures of solid organs. Three hollow anatomical architectures were chosen: the upper trachea, the urinary bladder, and the four-chamber heart. Open source CAD models were downloaded and modified for printing plastic templates with the intended anatomical geometries (Figure 4A–C).

Water-soluble polymer-based templates were then created through the Sof-T protocol as previously outlined (Figure 4D–F). The surface patterning features of the upper trachea, including the cartilaginous ring ridges of the tracheal wall, were well replicated in the alginate model (Figure 4G). The alginate-based scale-reduced bladder model replicated well the internal spheroidal geometry, as well as the protrusions of the ureters present in the printed template (Figure 4H), being also sufficiently mechanically robust to be manipulated by hand (Supplementary Figure S1A).

Similarly, the alginate-based heart construct (intentionally excluding the aortic root by submerging in alginate hydrogel only the heart proper) replicated the external geometry of the heart, although could not so far render all thin features of internal chambers (Figure 4I). As expected in this case, when compared to smaller models, this model took longer (>24 h) to dissolve, due to the limited surface area of the polymer scaffold exposed to water. Even so, the resulting alginate-based heart construct could be manipulated by hand without deformation (Supplementary Figure S1B).

Significantly, the total fabrication time required to produce the two hydrogel organ models was less than needed to produce similar geometries using layer-by-layer extrusion [30,36], or Digital Light Processing bioprinting [37] (Supplementary Table S1).

## 4. Discussion

***Advantages of the Soft Templating Approach.*** The soft templating method offers several distinct advantages over conventional bioprinting approaches for creating hollow hydrogel structures (Supplementary Table S1; see also [38]). Most significantly, this method is a form of ‘external molding’ particularly suitable to transfer anatomically realistic geometries from solid to soft materials such as hydrogels, which are notoriously difficult to do even by bioprinting.

The accessibility of required equipment represents another major advantage of this approach. While specialized bioprinters are expensive and require extensive training for effective operation [31,39], the Sof-T method requires only standard 3D printers that are widely available in academic, industrial, and even educational settings. This democratization of biofabrication capabilities could significantly expand the number of laboratories capable of producing complex tissue engineering constructs, potentially accelerating research progress across the field [40].

The speed and efficiency of the fabrication process also compare favorably to traditional bioprinting approaches. While bioprinting of complex structures can require 8–24 h to complete, depending on size and resolution requirements [30,36], the Sof-T method can produce similar constructs in 2–4 h once templates are prepared. This time efficiency is particularly advantageous for applications requiring large numbers of constructs or rapid prototyping of new designs.

Lastly, when used with hydrogel–cell mixtures, the Sof-T technique is expected to substantially reduce the risk of extrusion-associated shear stresses [28,41]. Traditional bioprinting exposes the cells to mechanical forces during the extrusion process that can damage cellular membranes, alter gene expression patterns, and reduce overall cell viability by a range of 20–40% [29,42]. The Sof-T method circumvents this limitation entirely by forming the hydrogel matrix around a pre-existing template in a gentle, low-stress environment that preserves cellular integrity.

***Limitations and Areas for Improvement.*** Despite these advantages, the Sof-T method has inherent limitations that must be acknowledged and addressed in future developments. The current approach is restricted to water-soluble polymers for template fabrication, which limits the range of template materials and may constrain the achievable geometric complexity. Additionally, so far, we applied this method only to a physically crosslinkable hydrogel, although dual physical–chemical crosslinking systems, such as that offered by methacrylated alginate or others, offer pathways for expanding material options (see also below) and improving mechanical properties [41,42].

Template removal from complex internal cavities also presents practical challenges that become more pronounced with increasing complexity. While simple tubular structures can be easily dissolved, more complex geometries with narrow passages, undercuts, or intricate internal features require longer washing times and/or retain template residues that could interfere with the intended construct applications, by affecting its biocompatibility or mechanical properties [43]. However, the observation that templates with extended calcium chloride soaking appear to dissolve more quickly suggests that the optimization of pre-treatment conditions could improve removal efficiency.

Regarding the dimensional fidelity of the constructs, it should be noted that in Sof-T the CAD specifies the external surface of the polymeric template, which is faithfully retained by 3D printing (and thus the inner diameter of the hydrogel construct), but the actual thickness of the hydrogel layer is determined by additional factors such as the gel’s viscosity, time of incubation, and the astringent effect of crosslinking, which makes the size of the resulting object not directly comparable to that of its CAD.

Our current mechanical characterization was performed using a single-axis tensile setup available to us, which yielded maximum tensile force and elongation at failure. However, Young’s modulus, burst pressure, compressive behavior, cyclic durability, and suture retention, which would provide a more complete mechanical portrait of SofT-made constructs, requires an instrumentation that we did not have access to in the current work; however, we are committed to a more comprehensive mechanical characterization in a follow-up study, along the dimensional-fidelity quantification against CAD.

Moreover, because the paper does not include work with cells, our “cell-friendly” claim regarding reduced shear stress is a predicted advantage of the gentle processing conditions, but not yet an experimentally demonstrated one. For the same reason, viability during template dissolution, the effect of possible polymeric or ionic residues, nutrient and oxygen diffusion in the wall thickness range achieved, alginate degradation kinetics under culture conditions, and long-term dimensional and mechanical stability of the constructs in aqueous environments are all open questions that must be addressed before actual biomedical applications can be pursued.

***Anticipated Impact.*** The anatomically inspired constructs produced by the Sof-T method could have immediate applications in surgical training and pre-operative planning [44], where accurate reproduction of patient-specific anatomies is essential for optimizing surgical outcomes [45]. The ability to create models that exhibit realistic mechanical properties while maintaining accurate geometric features could significantly enhance the value of these training tools compared to current rigid plastic or rubber alternatives [46].

In research applications, these constructs could also provide platforms for studying fluid dynamics, drug delivery, and disease progression in controlled laboratory environments [47]. The vascular models could serve as valuable tools for investigating blood flow patterns, wall stress distributions, and the effects of geometric variations on hemodynamic parameters [48].

Although important challenges (such as vascularization, long-term mechanical stability, biodegradation, and regulatory approval), have not yet been addressed in this study, the potential for in vivo applications remains promising, particularly for tissue engineering where the biocompatible nature of alginate constructs could serve as temporary scaffolds for tissue regeneration, and may eventually be explored for regenerative medicine applications, understandingly only after extensive biological validation.

***Future Directions and Planned Developments.*** Several specific improvements are planned for the next phase of development. For example, better control over hydrogel layer thickness represents a significant area. The current method relies on diffusion-controlled crosslinking processes in adsorbed hydrogels, which can result in non-uniform wall thickness, particularly in complex geometries with varying local curvatures and surface areas [49]. The observed deformations of small details, such as between branches in the aneurysmal model, suggest that optimization of alginate concentration, crosslinking ion concentration, or processing time could yield more uniform and mechanically robust structures.

The implementation of dual crosslinking using alginate–methacrylate with both calcium ions and ultraviolet light could provide enhanced mechanical properties and potentially improved control over crosslinking kinetics [19,50]; alternatively, it becomes possible to incorporate the cells in a softer alginate matrix to grow, then increase mechanical robustness afterwards. This approach could also address current limitations related to wall thickness’ uniformity and overall structural robustness.

In this report, we only show alginate models, but this method could potentially be used with natural hydrogels such as gelatin crosslinkable with genipin (already shown to be chemically compatible with PVA [51]) or fibrin derived from fibrinogen by diffusive polymerization with thrombin or transglutaminase from porous PVA [52], extending the diffusive enzymatic strategy demonstrated by Kolesky et al. [53] for Pluronic-based sacrificial inks to water-soluble filament-printed templates.

The incorporation of cells into the hydrogel matrices will represent a critical next step toward creating functional tissue constructs. The gentle processing conditions of the Sof-T method should be particularly advantageous for maintaining cell viability during fabrication [41], though the optimization of cell seeding densities, culture conditions, and nutrient transport will be necessary [26].

Stepwise soaking protocols using hydrogels with different chemical or cellular compositions offer the potential for creating multi-layered constructs that more accurately recapitulate the complex architecture of native tissues [22]. This approach could enable the production of constructs with distinct functional layers, such as endothelial linings combined with smooth muscle cell and/or fibroblast layers for vascular applications [6].

The creation of other hollow organ constructs including GI models will demonstrate the broader applicability of the Sof-T method beyond cardiovascular applications [2]. Each of these organ systems presents unique geometric and functional requirements that will help to further refine and optimize the methodology.

## 5. Conclusions

We developed the Sof-T method as a simple yet efficient approach for creating complex, hollow hydrogel structures intended to address some of the key limitations of current biofabrication technologies. However, the mechanical, diffusion, degradation, and biological behavior of Sof-T constructs are hydrogel-specific and thus the properties discussed here apply only to the alginate system tested. Extension to gelatin, fibrin, methacrylated alginate, or composite systems are possible, but will require independent characterization. Important challenges remain, such as vascularization, long-term mechanical stability, biodegradation, and regulatory approval, which have not been addressed in this study. Even so, our method’s ability to produce anatomically relevant constructs without subjecting cells to harmful shear stresses could provide a valuable alternative method to extrusion bioprinting. This technique represents an advancement in also making bioprinting-like activity accessible to students, researchers, and clinicians, thus making anatomically inspired hydrogel constructs accessible to laboratories that do not possess a sophisticated biofabrication infrastructure.

## Data Availability

The original contributions presented in this study are included in the article/Supplementary Material. Further inquiries can be directed at the corresponding author.

## Supplementary Materials

The following supporting information can be downloaded at: https://www.mdpi.com/article/10.3390/bioengineering13070838/s1, Figure S1: Handling of Sof-T fabricated alginate constructs; Table S1: Qualitative comparison of extrusion bioprinting, SLA/light-based bioprinting, and Soft Templating (SofT) across key fabrication parameters.

## Abbreviations

The following abbreviations are used in this manuscript:

3D	Three-dimensional
BaCl_2_	Barium chloride
BVOH	Butene-diol vinyl alcohol copolymer
CaCl_2_	Calcium chloride
CAD	Computer-aided design
FDM	Fused deposition modeling
NEST3D	Negative Embodied Sacrificial Template 3D
PLA	Polylactic acid
PVA	Polyvinyl alcohol
Sof-T	Soft templating
*w*/*v*	Weight per volume
