# Supplementary material for: Accessible Biofabrication of Anatomically Inspired Hollow and Branched Hydrogel Constructs by Soft Templating (Sof-T)
Source: Bioengineering (Basel). 2026 Jul 21;13(7):838. doi: 10.3390/bioengineering13070838 (PMC13404534; doi:10.3390/bioengineering13070838)

Accessible Biofabrication of Anatomically Inspired Hollow and Branched Hydrogel Constructs by Soft Templating (Sof-T)

Jacob Dairaghi[1], Dominic Joseph[2], Horia J. Petrache[3]

and Nicanor I. Moldovan[4,*]

Supplementary Information

Supplementary General Sof-T Fabrication Protocol

The Sof-T method begins with the fabrication of water-soluble templates by any standard 3D printing technique, using available .stl files. We tested two hydrosoluble polymers, polyvinyl alcohol (PVA) and butene-diol vinyl alcohol copolymer (BVOH), as filaments on a commercial desktop 3D printer. PVA has a lower glass transition temperature and stronger hydrogen bonding than BVOH [1], meaning the polymer remains in a viscoelastic state longer before cooling. BVOH is a modified copolymer which reduces hydrogen bond interactions and solidifies more quickly which can improve dimensional accuracy and ease of use in 3D printing [2]. In addition, PVA has higher moisture sensitivity which can further reduce printing quality which further guided our decision to switch from the more common PVA to BVOH as a preferred water-soluble 3D printing filament.

Following template fabrication, the structures undergo an ion impregnation process designed to load them with crosslinking ions essential for subsequent hydrogel shaping. Templates are immersed in calcium chloride ($CaCl_2$) solution at 10% (w/v) concentration for about 10 minutes. In our experience, this soaking period allows for uniform distribution of divalent cations throughout the template structure and for maintaining sufficient mechanical integrity for subsequent processing steps, while the polymer starts becoming softer than in dry state (and from here the name of the method).

Alternatively, barium chloride ($BaCl_2$) solutions can be used, when stronger crosslinking - hence stiffer hydrogel - is desired, with the additional benefit of increased X-ray absorbency if the sample is intended to be imaged by microCT, as we have shown elsewhere [3].

The ion-loaded templates are then immersed in diffusion-crosslinkable hydrogels, such as alginate solutions. the wall thickness of the resulting construct directly correlates with the alginate viscosity, allowing for controlled manipulation of its structural properties. High concentration alginate naturally results in thicker walls, thus providing a tunable parameter for optimizing mechanical characteristics based on intended applications.

Upon removal from the alginate solution, templates may be briefly returned to the crosslinking bath for several seconds to ensure complete crosslinking of the outer hydrogel layer. Following crosslinking, excess material is trimmed from the construct's ends, and if needed any other improperly formed sections to ensure uniform quality.

Template removal represents the final critical step in the fabrication process. For constructs with straight geometries, scaffolds can be directly extracted with a forceps. However, for complex geometries, including bifurcations and intricate internal structures, the templates are dissolved through immersion in

aqueous solutions such as water, phosphate buffered saline (PBS), or cell culture media. The dissolution process is doable at room temperature (25°C) but is faster at warmer temperatures (37°C) and with continuous stirring. While dissolution time changes based on exposed surface area and size of the template, full dissolution generally occurs within 1-3 hours for constructs on the scale of 1-5cm.

As a quick troubleshooting guide, we are suggesting the following solutions:

Problem	Likely Cause	Solution
Construct collapses	Alginate concentration too low	Increase concentration
Irregular wall thickness	Excess adsorption time	Reduce immersion
Template dissolves slowly	Low exposed surface area	Increase temperature/stirring
Poor geometric fidelity	Highly viscous alginate	Reduce concentration

Supplementary References

1. Tsioptsias, C.; Fardis, D.; Ntampou, X.; Tsivintzelis, I.; Panayiotou, C. Thermal Behavior of Poly(vinyl alcohol) in the Form of Physically Crosslinked Film. *Polymers (Basel)* **2023**, *15*.
2. Xing, J.; Wang, R.; Sun, S.; Shen, Y.; Liang, B.; Xu, Z. Morphology and Properties of Polylactic Acid Composites with Butenediol Vinyl Alcohol Copolymer Formed by Melt Blending. *Molecules* **2023**, *28*.
3. Dairaghi, J.; Benito Alston, C.; Cadle, R.; Rogozea, D.; Solorio, L.; Barco, C.; Moldovan, N.I. A Dual Osteoconductive-Osteoprotective Implantable Device for Vertical Alveolar Ridge Augmentation. *Frontiers in Dental Medicine* **2023**, *3:1066501*.

Supplementary Table S1

Qualitative comparison of extrusion bioprinting [4], SLA/light-based bioprinting [4], and Soft Templating (SofT) across key fabrication parameters

Method	Fabrication Speed	Build Volume	Gravity-Independent Fabrication	Monolithic Structure	Estimated Cost	Preferred Use Cases
**Extrusion Bioprinting**	Slow	128 × 85 × 60 mm (BioX)	Only with embedded/support-bath printing (e.g., FRESH)	Layer-by-layer	~$40,000 (BioX)	Multi-material/multi-cell construct simple geometries, high cell-densit bioinks
**SLA / Light-Based Bioprinting**	Medium	128 × 85 × 60 mm (LumenX)	Capable	Layer-by-layer	~$30,000–40,000 (LumenX)	High resolution, low cell density, s complex geometries, unsupported/ floating features
**Soft Templating (SofT)**	Fast	220 × 220 × 250 mm (Ender 3)	Capable	Monolithic	<$500	Low cost, large volume, fast gener of hollow geometries and monolith structures

4. Panja, N.; Maji, S.; Choudhuri, S.; Ali, K.A.; Hossain, C.M. 3D Bioprinting of Human Hollow Organs. AAPS *PharmSciTech* **2022**, *23, 139*.

Supplementary Figure S1

Handling of Sof-T fabricated alginate constructs: (**A**) bladder and (**B**) heart alginate models retained external anatomic structure during manual manipulation (useful for teaching and/or surgery planning applications). Scale bars: 1 cm.

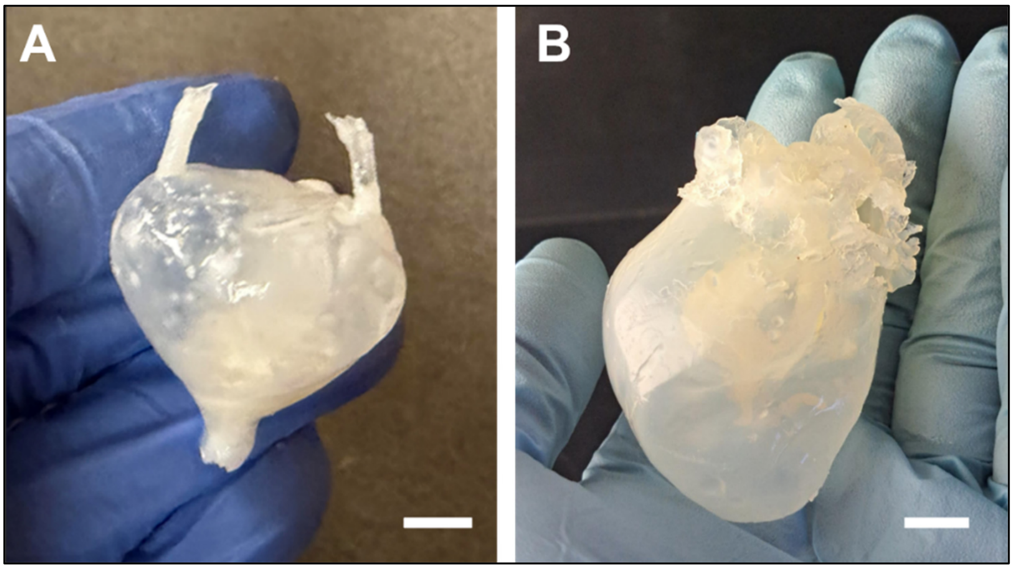

Supplement: Supplementary file 1 [file bioengineering-13-00838-s001.zip › bioengineering-4425118-supplementary.pdf]
